# Rate-adaptive pacing in heart failure with preserved ejection fraction: Too much of a good thing?

**DOI:** 10.1016/j.hroo.2024.03.010

**Published:** 2024-05-20

**Authors:** Alireza Oraii, Corentin Chaumont, Francis E. Marchlinski, Matthew C. Hyman

**Affiliations:** Cardiac Electrophysiology, Division of Cardiovascular Medicine, Department of Medicine, Hospital of the University of Pennsylvania, Philadelphia, Pennsylvania

**Keywords:** Diastolic dysfunction, Heart failure, Heart failure with preserved ejection fraction, Heart rate, Pacing, Sinus node modulation, Conduction system pacing, Exercise intolerance, Chronotropic incompetence, Pacemaker

**Key Findings**

▪ Rate-adaptive atrial pacing does not benefit heart failure with preserved ejection fraction (HFpEF) patients as assessed by exercise performance and health status, despite successful augmentation of heart rate during exercise.
▪ The complexity of cardiovascular and hemodynamic response to exercise may explain why some preclinical pacing studies have failed to translate to patients with HFpEF.
▪ Current evidence does not support pacemaker implantation in HFpEF patients beyond traditional pacemaker indications such as symptomatic bradycardia or conduction disease.

More than 64 million individuals worldwide and more than 6 million patients in the United States have been diagnosed with heart failure (HF).^1^^,^^2^ Heart failure with preserved ejection fraction (HFpEF) accounts for approximately half of HF cases and is anticipated to be the most common HF phenotype in the near future.^3^^,^^4^ Although many HFpEF patients feel comfortable at rest, they frequently have profound limitations in exercise capacity, cardiac output reserve, and notably blunted heart rate response to exertion.^5^^,^^6^ Whether sinus node dysfunction itself drives limitations in exercise capacity or is one marker out of many pathophysiologic abnormalities is uncertain.^7^ Chronotropic incompetence can significantly reduce cardiac output, especially during exercise when increased heart rate is required for maintaining sufficient peripheral oxygen delivery. Therefore, it is hypothesized that an augmentation in heart rate during exertion as a determinant of cardiac output may improve exercise capacity and quality of life in patients with HFpEF. The related question of whether heart rate augmentation via cardiac pacing can alleviate these symptoms has remained a persistent question in the field of HFpEF. Despite several studies to date, *there are inadequate data* *supporting* *pacemaker implantation to facilitate rate-adaptive pacing in HFpEF patients*.

Studies exploring pharmacologic slowing of sinus node function have demonstrated inconsistent findings when it comes to exercise capacity in HFpEF. Ivabradine selectively slows sinus rates through inhibition of I_f_ currents in the sinus node. Small randomized studies exploring the short-term impact of ivabradine on exercise capacity in patients with HFpEF have shown mixed results. Whereas one placebo-controlled, parallel-design randomized trial showed an increase in exercise capacity and peak oxygen consumption (VO_2_) after 7 days, a second placebo-controlled, crossover randomized trial showed worsening of peak VO_2_ and exercise capacity after 2 weeks of treatment with ivabradine.^8^^,^^9^ These conflicting findings could have been due to differences in baseline characteristics of study participants and short duration of follow-up. In contrast, beta-blocker therapy, previously a central strategy in HFpEF management, has more consistently been shown to adversely impact aerobic capacity and exercise tolerance in patients with HFpEF.^9^^,^^10^ Discontinuation of beta-blocker therapy improves functional capacity and chronotropic incompetence in HFpEF patients.^11^ Notably, beta-blockers are more pleiotropic in nature when compared to ivabradine. While heart rate reduction is a marker of beta-blocker effect, their cardiovascular effects extend much further leading to changes in adrenergic tone as well as altered dynamics of cardiac relaxation and contractility.

**Figure 1:**
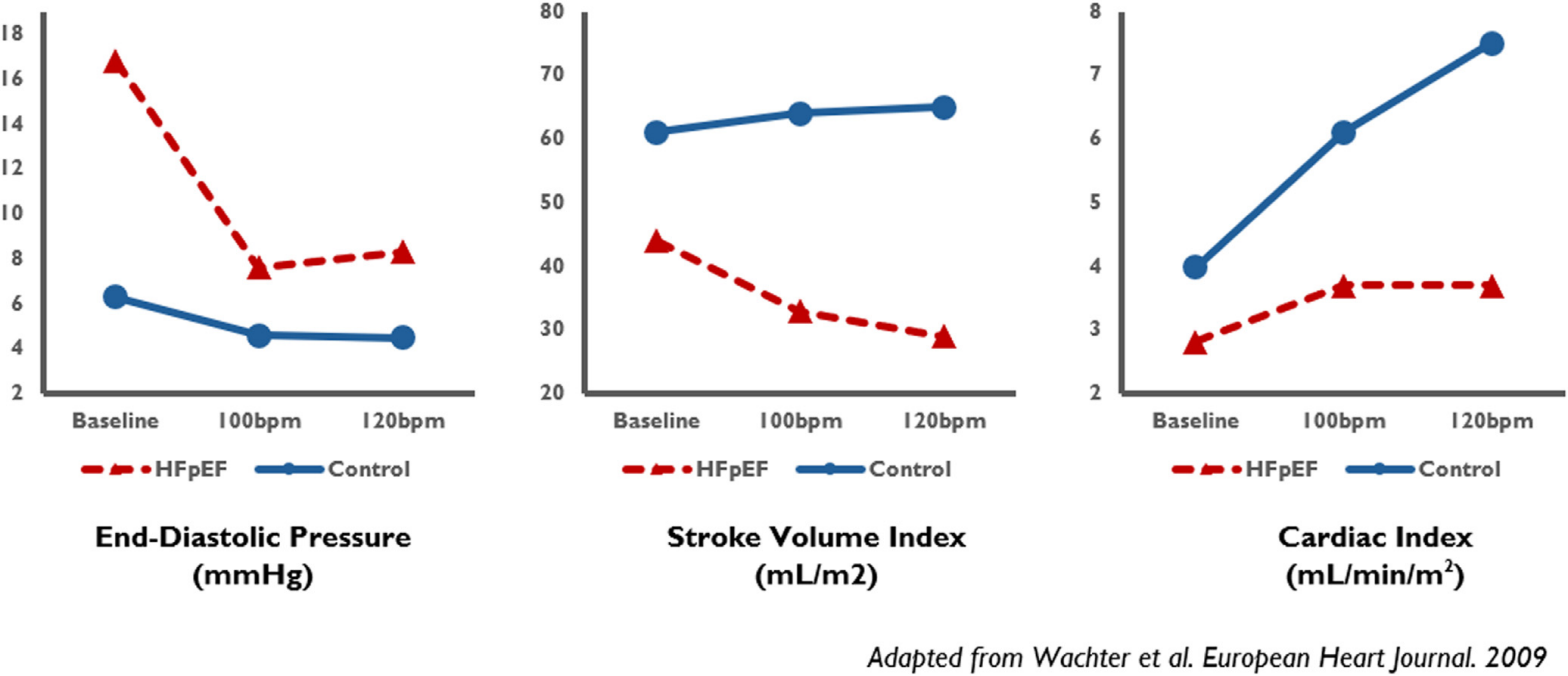
Cardiac hemodynamic changes at baseline and accelerated atrial pacing. HFpEF = heart failure with preserved ejection fraction.

Preclinical studies of atrial pacing in HFpEF may represent an overly simplistic model of adaptive heart rate changes during exercise. Preclinical studies have examined atrial pacing as a therapeutic intervention due to the strong associations of chronotropic incompetence with decreased aerobic capacity and cardiac output reserve in HFpEF patients.^5^ In healthy controls as well as in HFpEF patients, isolated atrial pacing at moderately elevated rates leads to reductions in left ventricular filling pressures.^12^^,^^13^ This effect is particularly pronounced in the HFpEF population, thereby proposing a new potential management target. With higher rates of atrial pacing, cardiac output initially increases in both normal and HFpEF groups before quickly reaching a plateau in the HFpEF population (Figure 1).^14^ Whereas contraction–relaxation dynamics in normal hearts can maintain cardiac output during exercise or accelerated atrial pacing, HFpEF patients may exhibit blunted pressure–volume changes at higher paced rates.^12^^,^^15^ The reasons for this are many, but patients with HFpEF are commonly found to have noncompliant left ventricles with elevated filling pressures, restricted ventricular relaxation, and increased wall stress.^16^ Therefore, the anticipated positive effect of increased heart rate on cardiac output potentially can be counterbalanced by a greater drop in stroke volume in patients with HFpEF.^17^ Although the tipping point above which an accelerated heart rate would paradoxically reduce cardiac output varies from patient to patient, those with HFpEF are hemodynamically susceptible because of their poor ventricular compliance and impaired relaxation during accelerated atrial pacing. However, one key limitation of atrial pacing studies is that they explore the impact of chronotropic incompetence in isolation. Cardiac hemodynamics during exercise are much more complex than alterations seen during the resting state, and exercise intolerance in HFpEF patients may not be fully explained by changes in central hemodynamic parameters alone. The above studies were unable to replicate how heart rate changes interact with the broader cardiovascular changes associated with exercise, including increased vascular tone, increase venous return, and changes in myocardial contractility and lusitropy in both normal and HFpEF populations.

**Figure 2:**
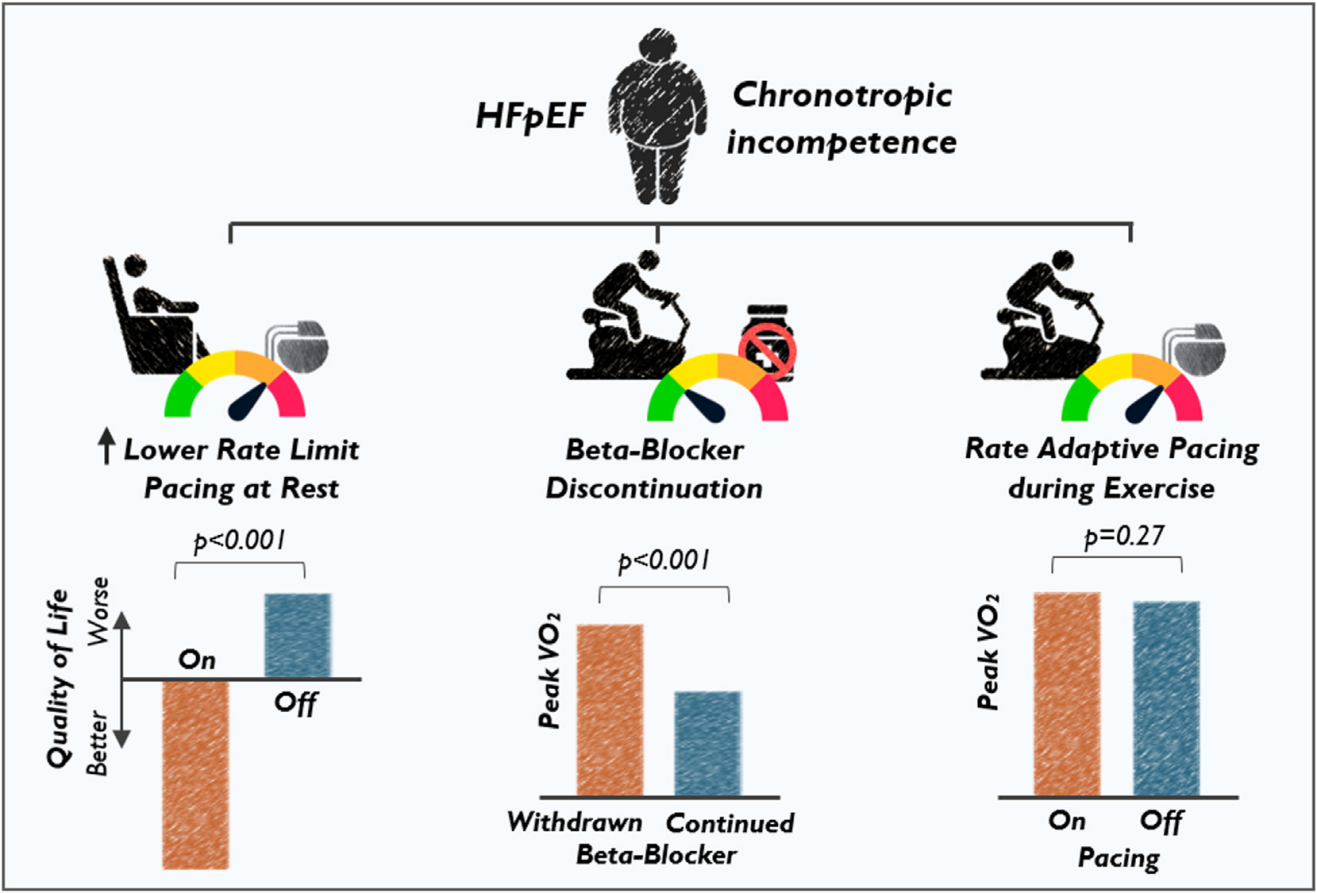
Heart rate modulation therapies in patients with heart failure with preserved ejection fraction (HFpEF) and chronotropic incompetence.

Randomized clinical trial data have failed to demonstrate a benefit to rate-adaptive pacing in HFpEF patients (Figure 2). The strongest data to date exploring the intersection of heart rate modulation with exercise capacity in the HFpEF population come from the recently published RAPID-HF (Rate-Adaptive Atrial Pacing in Diastolic Heart Failure) study.^18^ The RAPID-HF study was a single-center, double-blind, randomized crossover study investigating the role of rate-adaptive pacing in 32 patients with chronotropic incompetence and HFpEF. Notably, this study explored *de novo* implantation of pacemakers in patients with poor chronotropic reserve, though not an independent pacing indication. Clinical endpoints were focused on objective physiologic outcomes assessed by cardiopulmonary exercise stress testing. Despite preclinical data suggesting a possible benefit, the RAPID-HF trial showed that rate-adaptive pacing during exercise did not result in a significant improvement in the primary endpoint of change in VO_2_ at anaerobic threshold (absolute difference 0.3 mL/kg/min; 95% confidence interval [CI] −0.5 to 1.0 mL/kg/min; *P* = .46). Consistent with this finding, secondary endpoints exploring exercise capacity measured by peak VO_2_ (absolute difference 0.4 mL/kg/min; 95% CI −0.4 to 1.2; *P* = .27), quality of life (change in Kansas City Cardiomyopathy Questionnaire Overall Summary Score −0.9; 95% CI −11.0 to 9.3; *P* = .86) failed to demonstrate a clinical benefit as well.

It is possible the RAPID-HF study results were impacted by the degree to which heart rate was augmented during the rate-adaptive pacing “on” portion of the study. Rate-adaptiveness sensor sensitivity was adjusted on an individual basis to achieve heart rates of 100–110 bpm with brisk walking independent of other hemodynamic considerations. Although this strategy was successful as heart rates were significantly augmented (∼15 bpm) with exercise during the rate-adaptive pacing “on” arm of the study, this heart rate change was associated with a reduction in peak exercise stroke volume with accelerated pacing (absolute difference −24 mL; 95% CI −43 to −5 mL; *P* = .02), potentially impacting gains in peak exercise cardiac output (absolute difference −0.7 L; 95% CI −1.7 to 0.3 L/min; *P* = .14).

A second major pacing trial in HFpEF, *my*PACE, tested a method of device programming in patients with a pre-existing pacemaker indication, not whether HFpEF itself warranted pacemaker implantation (Figure 2). It should be noted that *my*PACE contrasts with RAPID-HF by demonstrating a benefit of increased resting lower rate limit pacing.^19^ In a randomized controlled trial of HFpEF patients with a pre-existing pacemaker indication (sinus node dysfunction), investigators showed a significant improvement in quality of life, increased physical activity, and cardiac biomarkers by programming the lower rate limit to 70–75 bpm in contrast to the traditional 60 bpm. The findings of RAPID-HF and *my*PACE are not necessarily conflicting because they test fundamentally different questions with inherently different study designs, patient populations, and trial endpoints. RAPID-HF assessed the role of atrial rate-adaptive pacing during submaximal and peak exercise in HFpEF, while *my*PACE assessed the role of moderately accelerated lower rate limit pacing during a resting state independent of rate-adaptive response. These studies should be viewed as complementary and in some cases confirmatory. A prespecified subanalysis of *my*PACE failed to demonstrate a benefit to rate-adaptive pacing beyond what was observed with increasing the lower rate limit, consistent with the RAPID-HF study. That said, patients in *my*PACE were a decade older, had more comorbidities (eg, atrial fibrillation), had higher N-terminal pro–brain natriuretic peptide levels, and most importantly had a pre-existing pacemaker indication for manifest sinus node dysfunction. In contrast, *de novo* experimental pacemaker implantation was required after enrollment for low heart rate reserve in RAPID-HF. Lastly, the 2 trials used different endpoints, including clinical outcomes (quality-of-life scores) over 1-year follow-up in *my*PACE vs physiologic assessments (VO_2_ testing) after 4 weeks in RAPID-HF. These differences suggest the presence of a more advanced substrate manifesting as more severe sinus node dysfunction in the *my*PACE compared to the RAPID-HF study population that may have enriched for those patients receiving a pacing benefit. This as well as changes in left atrial pressure at rest may explain some of the observed quality-of-life benefits and increase in physical activity with accelerated pacing at resting state during follow-up.

Although both trials provide uniquely important findings about the role of pacing in patients with HFpEF, the differences noted indicate that each trial should be interpreted within its own clinical context. As a final point, it should be noted that both studies were cautious to use atrial pacing in patients with an intact conduction system or conduction system–based ventricular pacing to avoid pacing-induced ventricular dyssynchrony.

Pathologic exercise responses in HFpEF patients are multifactorial and will be difficult to fix with tools targeting a single aspect of a maladaptive exercise response. The most effective therapies in HFpEF to date have been broad in their biological impact. Sodium glucose cotransporter-2 inhibitors and glucagonlike peptide-1 agonists have the clearest data in large clinical trials, showing promising effects in patients with HFpEF with respect to mortality benefit, and improvements in quality of life, physical limitation, and functional capacity.^20^^,^^21^ However, their effects are thought to be mediated through a diversity of pathways including blood pressure lowering, weight loss, and improving renal function amongst others.^21^, ^22^, ^23^ Other examples of therapies targeting individual pathobiological responses in HFpEF have been less successful. For example, elevated left ventricular end-diastolic pressure and the resulting increase in pulmonary capillary wedge pressure is thought to be a limiting factor in exercise capacity in patients with HFpEF. A recent crossover randomized trial showed that nitroglycerin-induced reduction in pulmonary capillary wedge pressure during exercise did not improve peak oxygen uptake in patients with HFpEF.^24^ This finding not only argues against the role of high filling pressures as the sole limiting factor for exercise intolerance but further supports a multisystem pathophysiology in patients with HFpEF involving cardiovascular, pulmonary, endothelial, and systemic metabolic abnormalities.^7^^,^^25^, ^26^, ^27^ Peripheral skeletal abnormalities in oxygen utilization as reflected by reduced exercise arteriovenous oxygen difference also indicate the important contributing role of noncardiac factors to exercise intolerance in patients with HFpEF.^10^ These concepts accentuate the need for a holistic approach in the management of patients with HFpEF utilizing lifestyle risk factor modification, pharmacologic interventions, and, in certain scenarios, device therapies.

In summary, the multifactorial nature of HFpEF suggests that chronotropic incompetence associated with exercise is a symptom of a disease process more than the root of the problem. Randomized trial data of rate-adaptive pacing in the HFpEF population have failed to demonstrate a clinical benefit in cardiopulmonary exercise testing. Overall, there may be a benefit in increasing lower heart rate limit in symptomatic patients with sinus node dysfunction and HFpEF, but the therapeutic window likely is narrow during exertion, if not completely blunted. With the current data available, there is no consistent and therefore convincing evidence for pacemaker implantation to facilitate rate-adaptive pacing in the HFpEF population.
